# A Shared Genetic Propensity Underlies Experiences of Bullying Victimization in Late Childhood and Self-Rated Paranoid Thinking in Adolescence

**DOI:** 10.1093/schbul/sbu142

**Published:** 2014-10-16

**Authors:** Sania Shakoor, Phillip McGuire, Alastair G. Cardno, Daniel Freeman, Robert Plomin, Angelica Ronald

**Affiliations:** ^1^Department of Psychological Sciences, Centre for Brain and Cognitive Development, Birkbeck, University of London, UK;; ^2^Department of Psychosis Studies, Institute of Psychiatry, King’s College London, UK;; ^3^Academic Unit of Psychiatry and Behavioral Sciences, University of Leeds, UK;; ^4^Department of Psychiatry, University of Oxford, UK;; ^5^MRC Social, Genetic & Developmental Psychiatry Centre, Institute of Psychiatry, King’s College London, UK

**Keywords:** bullying victimization, psychotic experiences, twin study, paranoia

## Abstract

*Background:* Bullying is a risk factor for developing psychotic experiences (PEs). Whether bullying is associated with particular PEs, and the extent to which genes and environments influence the association, are unknown. This study investigated which specific PEs in adolescence are associated with earlier bullying victimization and the genetic and environmental contributions underlying their association. *Method:* Participants were 4826 twin pairs from a longitudinal community-based twin study in England and Wales who reported on their bullying victimization at the age of 12 years. Measures of specific PEs (self-rated Paranoia, Hallucinations, Cognitive disorganization, Grandiosity, Anhedonia, and parent-rated Negative Symptoms) were recorded at age of 16 years. *Results:* Childhood bullying victimization was most strongly associated with Paranoia in adolescence (*r* = .26; *P* < .01), with weaker associations with Hallucinations, Cognitive Disorganization, parent-rated Negative Symptoms (*r* = .12–.20; *P* < .01), Grandiosity (*r* = .04; *P* < .05), and Anhedonia (*r* = .00, n.s.). Bivariate twin model-fitting demonstrated that bullying victimization and Paranoia were both heritable (35% and 52%, respectively) with unique environmental influences (39% and 48%, respectively), and bullying victimization showed common environmental influences (26%). The association between bullying victimization and Paranoia operated almost entirely via genetic influences (bivariate heritability = 93%), with considerable genetic overlap (genetic correlation = .55). *Conclusion:* In contrast to the assumed role of bullying victimization as an environmental trigger, these data suggest that bullying victimization in late childhood is particularly linked to self-rated Paranoia in adolescence via a shared genetic propensity. Clinically, individuals with a history of bullying victimization are predicted to be particularly susceptible to paranoid symptoms.

## Introduction

Epidemiological studies have shown that exposure to childhood adversities (bullying, physical abuse, sexual abuse, and maltreatment) increases the risk of developing psychotic experiences (PEs)^1–4^ and psychotic disorders.^5^ As a prevalent form of childhood adversity affecting ~13% of children and adolescents worldwide,^6^ being a victim of bullying has been associated with PEs. Bullied children are at an increased risk of developing adjustment problems (ie, anxiety, depression, suicidal ideation)^7^ and are at an approximate 2- to 4-fold increased risk of having PEs (ie, Hallucinations, Delusions, and Paranoia).^2,4,8^ These associations are robust and independent of the effects of psychopathology, family adversity, family psychiatric history, and IQ.^2,4,8^ Victims of bullying are therefore a vulnerable group of young people at risk of developing PEs. However, not all bullied children and adolescents go on to develop PEs, raising the question as to what determines whether exposure to bullying victimization will lead to psychopathology in later life. Research into resiliency and protective factors suggests school and family factors (ie, maternal warmth and positive atmosphere) have a protective effect against the developmental of adjustment problems amongst victims of bullying.^9^ It is possible that these factors may also extend to the risk of PEs. Because some of these factors (ie, maternal warmth in parenting) are in part heritable,^10^ investigations into why some victims of bullying develop PEs could benefit from starting with the examination of genetic and environmental contributions to the association between bullying victimization and PEs. The existing literature has largely focused on PEs in general and has not tested whether bullying increases the risk of specific types of PE. An improved understanding of how individual differences in bullying victimization contributes to the risk of PEs is imperative because it could not only help a group of vulnerable individuals who are at risk of experiencing difficulties in later life,^7^ but also help target factors which increase vulnerabilities to PEs.

Individual differences in experiencing bullying victimization are both genetically and environmentally influenced,^11^ as are PEs.^12–15^ Assuming bullying victimization is a purely “environmental” trigger for PEs may therefore be misguided. A genetically-sensitive design is needed to disentangle what causes bullying victimization to predict PEs. In a study of young adolescents, researchers composed an index of genetic risk as a function of zygosity and cotwins’ levels of psychotic symptoms. Bullying victimization between 5 and 12 years was a risk factor for PEs in young adolescents, independent of their genetic risk.^2^ Although this study accounted for genetic risk it did not estimate the extent to which genetic factors contributed to the covariance between bullying victimization and PEs. Furthermore, it only assessed 2 types of PEs: Hallucinations and Delusion. Principal component and factor analyses suggest that multiple components underlie PEs.^16,17^

To our knowledge, this is the first study to assess formally the degree of genetic and environmental influences contributing to the association between bullying victimization and specific PEs (including a range of positive, cognitive, and negative experiences) within the general population. In particular, we were interested in investigating bullying victimization at age 12 years because making the transition from primary to secondary school can be a vulnerable time for children and can often results in changes in peer groups. Adolescents commonly report and feel distressed by PEs^18^ but have not yet reached the age when psychotic disorders typically are diagnosed. As such, it is an important developmental stage to capture the causes and predictors of PEs in the general population. This study aimed to examine if bullying victimization in late childhood was associated with specific PEs in adolescence and estimate the extent to which genetic and environmental factors influenced the association between bullying victimization and different forms of PEs. Our first hypothesis was that bullying victimization would be differentially associated with particular types of PE. Cognitive psychological theories of the development of PEs^19^ suggest that exposure to “triggering events” (ie, bullying victimization) are particularly damaging in individuals predisposed to disruptions in their cognitive processes, which in turn may contribute to their risk for positive PEs. Because there is also evidence to suggest that bullied children and young adults are more likely to have disruptions in their cognitive processes such as negative attributional styles,^20,21^ we hypothesized that bullying victimization would be strongly associated with positive PEs. We then tested the hypothesis that bullying is not a purely environmental risk factor for PEs, but that there is also a genetic component to its effects.

## Methods

### Sample

Participants were members of the Twins Early Development Study (TEDS), a general population sample of monozygotic (MZ) and dizygotic (DZ) twins born in England and Wales between 1994 and 1996 and assessed longitudinally across childhood and adolescence.^22^ The TEDS has full ethical approval and written consent was obtained at point of contact.

At the age of 12 years, 8438 families from TEDS were contacted to take part by completing web-based tests and questionnaire interviews. Parent reports for 5854 (69%) families and twin reports for 5858 (69%) pairs were obtained. Participating children had a mean age of 11.56 years. Individuals were excluded (*N* = 337 families) if they did not provide consent at first contact (when TEDS was started), if they had severe medical disorder, had experienced severe perinatal complications, or if their zygosity was unknown. After exclusions data was available from 4972, among whom 44% were male and 37% were MZ twin pairs.

At the age of 16 years, 10 874 families from TEDS were invited to take part in the Longitudinal Experiences And Perceptions (LEAP) study, which focuses on the causes of PEs in adolescence.^17^ Parent reports for 5076 (47%) families and twin reports for 5059 (47%) pairs were obtained. Adolescents involved in the LEAP project had a mean age of 16.32 years. Individuals were excluded (*N* = 436 families) if they did not provide consent at first contact (when TEDS was started), if they had severe medical disorder, had experienced severe perinatal complications, or if their zygosity was unknown. After exclusions, data was available from 4826 families (45% male, 36% MZ twin pairs).

### Measures

#### Bullying Victimization.

Bullying victimization was assessed at the age of 12 years using the Multidimensional Peer Victimization Scale,^23^ which has been shown to be a reliable and valid measure of bullying victimizations.^23^ The measure consisted of 16 items, which encompassed bullying behaviors such as physical abuse, verbal abuse, social manipulation, and property damage. Participants were asked to report on “*How often has another pupil done these things to you in the past school year*,” by responding “*Not at all*” (0), “*Once*” (1), “*More than once*” (2) to items such as “called me names,” “punched me,” “made fun of my appearance,” and “refused to talk to me.” Summing across all items captured a total composite measure of bullying victimization.

#### Psychotic Experiences.

Psychotic experiences were assessed using the Specific Psychotic Experiences Questionnaire (SPEQ).^17^ The SPEQ assesses specific PEs as quantitative traits and includes 5 self-report subscales: Paranoia (15 items), Hallucinations (9 items), Cognitive Disorganization (11 items), Grandiosity (8 items), Anhedonia (10 items), and one parent-rated subscale: parent-rated Negative Symptoms (10 items). The SPEQ items were derived for the most part from existing scales that were adapted to be suitable for adolescents.^17^ The subscales were derived from principal component analysis and show good-to-excellent internal consistency (*r* = .77–.93) and test–retest reliability across a 9-month interval (*r* = .65–.74) in this sample. In terms of validity, expert clinical opinion was obtained on the suitability of each item as a measure of adolescent PEs to ensure content validity.^17^ Furthermore, levels of agreement between scores on SPEQ and the PLIKS (a known measure of psychosis-like symptoms)^24^ showed that adolescents who reported “definitely” having any psychosis-like symptoms on the PLIKS had significantly more PEs on all the SPEQ subscales (with exception of Anhedonia) when compared with those who did not report any definite psychosis-like symptoms (all significant at *P* < .001). Positive and cognitive subscales of PEs showed significant positive correlations with the PLIKS quantitative score (Hallucinations *r* = .60, Paranoia *r* = .48, Cognitive Disorganization *r* = .41, Grandiosity *r* = .27, all *P*’s < .001).^17,24^ Further information on the measure can be found by Ronald and colleagues.^17^

#### Additional Measures.

Bullying victimization was measured at the age of 16 years using a shortened (6 items) version of the Multidimensional Peer Victimization Scale.^23^ Anxiety was measured at the age of 12 years using parent reports of the Strengths and Difficulties Questionnaire.^25^ Depression was measured at the age of 12 years using parent reports of the Moods and Feelings Questionnaire.^26^ Cannabis use was assessed at the age of 16 years by asking participants “*Have you ever tried cannabis*,” and personality was assessed using a self-report on a published scale^27^

### Statistical Analyses

All analyses were performed using STATA 12^28^ and OpenMx.^29^ OpenMx uses the method of maximum likelihood estimation, which is widely used for analyzing genetically sensitive data and deals with missing data. In line with standard behavioral genetics procedure, the effects of sex and age were regressed out, and analyses were conducted using standardized residuals.^30^ Scales of bullying victimization and PEs were transformed using square root transformation techniques to reduce skewness and kurtosis and to ensure that the assumption of having a normal distribution was met for genetic modeling. Analyses were performed in the following steps. First, the extent to which bullying victimization was associated with specific PEs in adolescence was assessed using Pearson’s correlations. Twin-model fitting was conducted for associations with a Pearson’s correlation >.25 because this was considered to be adequate phenotypic covariation to warrant twin model-fitting. Second, the degree of twin similarity on the measures was investigated using intraclass correlations separately for MZ and DZ groups. Univariate structural equation models were used to estimate the contributions of genetic and environmental influences on bullying victimization and specific PEs. Finally, bivariate twin models were run to test to what degree genetic and environmental influences on bullying victimization overlapped with genetic and environmental influences on specific PEs.

#### The Twin Design.

The twin design involves MZ and DZ twin pairs to determine the extent to which variation in a single phenotype, or covariation between phenotypes are attributable to genetic and environmental influences. Within pair similarities separately for MZ and DZ, twin pairs were examined to establish the role of genetic and environmental influences based on the notion that: (1) MZ twin pairs share 100% of their segregating DNA code and DZ twin pairs share on average 50%; (2) MZ and DZ twin pairs share environmental factors common to both twins in the same family (“common environment”); and (3) Exposure to environmental factors which are experienced differently or are specific to the individual (“unique environment”) contribute towards differences between MZ and DZ twin pairs.^31^

Structural equation modeling techniques were employed to establish the relative importance of additive genetic (A), common environment (C), and unique environmental influences (E) contributing to a phenotype.^31^ This technique further extends to bivariate analyses, by exploring the covariation between phenotypes. The relative contributions of genetic and environmental factors to the association between 2 phenotypes are referred to as bivariate heritability (biva2), bivariate common environment (bivc2), and bivariate unique environment (bive2). Estimates of covariance were also used to calculate genetic correlations (*r*_a_), common environment correlations (*r*_c_), and unique environment correlations (*r*_e_), which indexed the extent to which the same set of genes or environments influence both phenotypes.^32^ The relative fit of different models were compared to a saturated model (which provides a full description of the data) to establish the best fitting model for the data.^33^ Parameter estimates were then calculated with confidence intervals using the maximum-likelihood method. The best fitting models were selected on the basis of goodness of fit using the likelihood ratio test and the Bayesian Information Criterion (BIC).

## Results

Table 1 presents means and standard deviations for the 6 PEs scales, and bullying victimization for each gender and zygosity group. Analyses of variances (ANOVA) illustrated significant main effects of gender for all of the PEs scales. Relative to males, females reported higher levels of Paranoia, Hallucinations, and Cognitive Disorganization. In contrast, males reported higher levels of Grandiosity, Anhedonia, and had more parent-rated Negative Symptoms when compared with females. There was an effect of gender for bullying victimization, whereby males reported being bullied more than females. A main effect for zygosity was observed for Paranoia, Hallucinations, Cognitive Disorganization, and parent-rated Negative Symptoms, whereby DZs reported higher levels in comparison to MZs. However, the combined effect of gender and zygosity on the means was small (*R* ^2^ = .00–.06).

**Table 1.** Means, Standard Deviations and Analysis of Variance by Sex and Zygosity for Psychotic Experiences and Bullying Victimization

	Total	Male	Female	MZ	DZ	Score	Skew	Kurtosis	Cronbach α	ANOVA				
	*M* (*SD*)	*M* (*SD*)	*M* (*SD*)	*M* (*SD*)	*M* (*SD*)	Range				Sex	Zyg	Sex*Zyg	*R* ^2^	*N*
Psychotic experiences														
Paranoia	12.17 (10.62)	11.75 (10.42)	12.50 (10.77)	11.79 (10.46)	12.37 (10.70)	0–71	0.17	3.07	.93	<0.01	0.01	0.45	.00	4.776
Hallucinations	4.65 (6.00)	4.30 (5.77)	4.94 (6.16)	4.47 (5.91)	4.76 (6.05)	0–45	0.55	2.74	.87	<0.01	0.01	0.53	.01	4.784
Cognitive Disorganization	3.96 (2.85)	3.40 (2.72)	4.41 (2.87)	3.86 (2.82)	4.01 (2.86)	0–11	0.44	2.31	.73	<0.01	0.01	0.66	.03	4.777
Grandiosity	5.32 (4.42)	5.82 (4.56)	4.91 (4.27)	5.26 (4.35)	5.35 (4.46)	0–24	−0.08	2.79	.85	<0.01	0.56	0.96	.01	4.780
Anhedonia	17.33 (7.93)	19.50 (7.98)	15.58 (7.44)	17.07 (7.96)	17.48 (7.91)	0–50	−0.48	3.10	.78	<0.01	0.44	0.85	.06	4.780
Parent-rated Negative Symptoms	2.81 (3.89)	3.17 (4.10)	2.52 (3.69)	2.64 (3.57)	2.91 (4.06)	0–30	0.56	2.72	.85	<0.01	0.03	0.02	.01	4.792
Bullying victimization	7.55 (7.24)	8.40 (7.63)	6.82 (6.79)	7.61 (7.37)	7.45 (7.11)	0–32	0.03	2.23	.91	<0.01	0.54	0.22	.01	3.884

*Note*: Means and *SD* reported prior to transformation. DZ = dizygotic twins; MZ = monozygotic; *SD* = standard deviation. Skew and kurtosis reported after transformation for normality. Analyses of variances were performed using 1 random member of each twin pair. Sex = *P* value associated with the effect of sex on the means; Zyg. = *P* value associated with the effect of zygosity on the means; Sex*Zyg = *P* value associated with the effects of the interaction between sex and zygosity on the means; *R* ^2^ = proportion of the total variance explained by sex and zygosity; *N* = number of randomly selected individuals from each twin pair.

Phenotypic correlations between bullying victimization and PEs are presented in table 2. Childhood bullying victimization was most strongly associated with Paranoia in adolescence (*r* = .26; *P* < .01), whereas associations were lower but significant with Hallucinations, Cognitive Disorganization, and parent-rated Negative Symptoms (*r* = .12–.18; *P* < .01), and barely present for Grandiosity (*r* = .04; *P* < .05), and Anhedonia (*r* = .00, n.s.).

**Table 2.** Phenotypic Correlations

Psychotic Experiences	Bullying Victimization at Age-12 Years
	*r* (CI)
Paranoia	.26 (0.23, 0.28)
Hallucinations	.18 (0.15, 0.20)
Cognitive disorganization	.20 (0.17, 0.22)
Grandiosity	.04 (0.01, 0.06)
Anhedonia	.00 (−0.02, 0.03)
Parent-rated Negative Symptoms	.12 (0.09, 0.15)

*Note*: Correlations were performed using 1 random member of each twin pair using standardized age and sex regressed residuals. *r* = Pearson’s correlation; CI = confidence intervals.

We performed behavior genetic twin analyses on the relationship of Paranoia with bullying victimization, in light of this relationship having a phenotypic correlation > .25.

### Genetic Analyses

For Paranoia and bullying victimization, univariate twin correlations (table 3) were indicative of genetic effects (A), because MZ correlations were larger than DZ correlations. Because the DZ correlations were greater than half of MZ correlations, this suggested some common environmental (C) influence on Paranoia and bullying victimization. Furthermore, because MZ correlations were less than unity, this implied a modest unique environmental effect (E) on Paranoia and bullying victimization. We observed little differences in twin correlations when split by sex (see supplementary table 1), thus supporting our decision not to split our analyses by sex to ensure maximum power to detect genetic and environmental influences.

**Table 3.** Intraclass Twin Correlations and Numbers of Participants

	MZ	DZ	Number of Participants				
	ICC (CI)	ICC (CI)	Males	Females	MZ	DZ	Pairs
Univariate twin correlations							
Paranoia	0.52 (0.49, 0.56)	0.29 (0.24, 0.33)	1378	1913	1719	1551	3268
Bullying victimization	0.62 (0.58, 0.65)	0.42 (0.37, 0.46)	1116	1569	1418	1258	3404
Cross-trait cross-twin correlation							
Paranoia and bullying victimization	0.26 (0.21, 0.31)	0.12 (0.07, 0.18)					

*Note*: Abbreviations are explained in the first footnote to tables 1 and 2. Intraclass correlations using transformed standardized age and sex regressed scales. ICC = intraclass correlations.

Univariate model fitting analyses confirmed initial observations from the twin correlations by showing that for both Paranoia and bullying victimization both genetic (52% and 35%, respectively) and unique environmental (48% and 39%, respectively) factors contributed most to the observed variance. Common environment had a significant influence on bullying victimization (26%) (table 4). Both univariate ACE models did not provide a significantly worse fit when compared with the saturated models. C explained a small amount of the variance for Paranoia (7%) and could be dropped from the model.

**Table 4.** Fit Statistics and Parameter Estimates for Best Fitting Univariate and Bivariate Models: Bullying Victimization and Paranoia

Univariate Mode1										
	Model Fit									
	Model	Compared to Saturated Model						Parameter Estimates		
		−2LL	*df*	LRT	Δ*df*	BIC	*P*	*A* (CI)	*C* (CI)	*E* (CI)
Paranoia	Sat	23507.87	6525	—	—	—	—	—	—	—
	ACE	23511.61	6531	3.75	6	−29396.49	.71	.45 (0.34, 0.54)	.07 (0.00, 0.16)	.48 (0.45, 0.52)
	CE	23580.53	6532	72.66	7	−29335.67	< .01	—	—	—
	AE^a^	23513.44	6532	5.57	7	−29402.76	.59	.52 (0.49, 0.55)	-	.48 (0.45, 0.51)
Bullying victimization	Sat	18060.00	5339	—	—	—	—	—	—	—
	ACE^a^	18064.96	5345	4.97	6	−28226.88	.55	.35 (0.25, 0.45)	.26 (0.16, 0.34)	.39 (0.36, 0.43)
	CE	18113.79	5346	53.79	7	−28186.71	< .01	—	—	—
	AE	18091.22	5346	31.22	7	−28209.28	< .01	—	—	—
Bivariate Mode1										
	Model Fit									
	Model	Compared to Saturated Model								
		−2LL	*df*	LRT	Δ*df*		BIC		*P*	
Paranoia	Saturated	31403.88	11856	—	—		—		—	
	ACE	31415.14	11873	11.26	17		−71414.23		.84	
	CE	31529.74	11876	125.86	20		−71325.61		<.01	
	AE	31443.96	11876	40.08	20		−71411.39		<.01	
	ACE dropped *r*_a_	31431.08	11874	27.20	18		−71406.95		<.01	
	ACE dropped *r*_c_^a^	31418.95	11874	15.07	18		−71419.08		.66	
	Parameter Estimates for Best Fitting Bivariate Model: Bullying Victimization									
	Best Fitting Model	Bivariate *a* ^2^	Bivariate *c* ^2^	Bivariate *e* ^2^	*r*_a_		*r*_c_		*r*_e_	
Paranoia	ACE dropped *r*_c_	.93 (0.85, 1.00)	—	.07 (−0.02, 0.15)	.55 (0.45, 0.70)		—		.04 (−0.01, 0.09)	

*Note*: ACE = full model testing genetic, common and unique environmental influences; AE = model testing genetic and unique environment influences; CE = model testing common and unique environmental influences; Sat= saturated model; −2LL = negative 2 log likelihood; *df* = degrees of freedom; LRT = likelihood ratio chi-square test comparing the −2LL fit of each model to the −2LL fit of the saturated model; Δ*df* = difference in degrees of freedom comparing each model to the saturated model; BIC = Bayesian Information Criterion (lower values reflect a better fit); *P* = *P* value. Bivariate genetic (Bivariate *a* ^2^), common environment (Bivariate *c* ^2^), and unique environment (Bivariate *e* ^2^) estimated indicate the proportion of phenotypic correlations explained by genetics, common, and unique environment, respectively. Bivariate genetic (*r*_a_), common environment (*r*_c_), and unique environment (*r*_e_) correlations indicate the genetic and environmental overlap between psychotic experiences and bullying victimization. 95% confidence intervals in parentheses.

^a^Best fitting model.

Bivariate cross-twin cross-trait (CTCT) correlations (table 3) provided an insight into the extent to which the covariance between bullying victimization and Paranoia was explained by genetic and common and unique environmental influences. MZ CTCT correlations were larger than DZ CTCT correlations for Paranoia and bullying victimization, which is indicative of genetic influences on the phenotypic association. MZ CTCT correlation was not less than the phenotypic correlation between bullying victimization and Paranoia, thus suggesting little to no unique environmental influence on the covariation.

Results from the bivariate correlated factors solution (table 4) showed that for the association between bullying victimization and Paranoia, the ACE with dropped *r*_c_ correlated factors solution fitted the data best based on the BIC fit index, whereby A, C, and E parameters were estimated for both phenotypes but the parameter estimating the correlation between Paranoia and bullying victimization attributable to common environmental influences was not set free to be estimated. Analyses demonstrated that the relationship between bullying victimization and Paranoia was almost completely explained by genetic influences (biva^2^ = 0.93), with the remaining covariance being explained by unique environment (bive^2^ = 0.07), although this was nonsignificant. The genetic correlation indicated that a considerable degree of genetic influences (*r*_a_ = .55) overlapped between bullying victimization and Paranoia. A small proportion of unique environmental (*r*_e_ = .04) overlap was also present; however, confidence intervals overlapped with 0.

Bivariate analyses adjusting for emotional problems (anxiety and depression) and bullying victimization at age of 16 years found ACE with dropped *r*_c_ correlated factors solution fitted the data best based on the BIC fit index (table 5). Similar findings were observed with genetic influences explaining most of the covariation between Paranoia and bullying victimization (emotional problems: 92%–93%; bullying victimization age of 16 years: 89%).

**Table 5.** Fit Statistics and Parameter Estimates for Best Fitting Bivariate Models: Bullying Victimization Age-12 and Paranoia Age-16 Adjusting for Emotional Problems at Age-12 and Bullying Victimization at Age-16

	Model Fit						
	Compared to Saturated Model						
	Model	−2LL	*df*	LRT	Δ*df*	BIC	*P*
Paranoia	Saturated	31403.88	11856	—	—	—	—
	ACE	31415.14	11873	11.26	17	−71414.23	.84
	CE	31529.74	11876	125.86	20	−71325.61	<.01
	AE	31443.96	11876	40.08	20	−71411.39	<.01
	ACE dropped *r*_a_	31431.08	11874	27.20	18	−71406.95	<.01
	ACE dropped *r*_c_^a^	31418.95	11874	15.07	18	−71419.08	.66
Paranoia controlling for anxiety	Saturated	31213.17	11805	—	—	—	—
	ACE	31224.85	11822	11.68	17	−71015.59	.82
	CE	31334.89	11825	121.71	20	−71052.78	<.01
	AE	31255.13	11825	41.96	20	−71158.52	<.01
	ACE dropped *r*_a_	31240.78	11823	27.61	18	−71172.87	.07
	ACE dropped *r*_c_^a^	31227.21	11823	14.03	18	−71169.12	.73
Paranoia controlling for depression	Saturated	31100.27	11803	—	—	—	—
	ACE	31113.73	11820	13.46	17	−71256.62	.70
	CE	31218.42	11823	118.15	20	−71177.91	<.01
	AE	31143.77	11823	43.50	20	−71252.56	<.01
	ACE dropped *r*_a_	31127.14	11821	26.87	18	−71251.87	.08
	ACE dropped *r*_c_^a^	31115.63	11821	15.36	18	−71263.38	.64
Paranoia controlling for anxiety and depression	Saturated	31097.23	11803	—	—	—	—
	ACE	31110.65	11820	13.42	17	−71259.70	.71
	CE	31214.94	11823	117.71	20	−71181.39	<.01
	AE	31141.22	11823	43.99	20	−71255.11	<.01
	ACE dropped *r*_a_	31124.22	11821	26.99	18	−71254.79	.08
	ACE dropped *r*_c_^a^	31112.45	11821	15.23	18	−71266.56	.65
Paranoia controlling for bullying victimization age-16	Saturated	20767.73	8026	—	—	—	—
	ACE	20779.56	8043	11.82	17	−48879.04	.81
	CE	20849.26	8046	81.52	20	−48835.33	<.01
	AE	20805.97	8046	38.24	20	−48878.62	<.01
	ACE dropped *r*_a_	20782.30	8044	14.56	18	−48884.97	.69
	ACE dropped *r*_c_^a^	20779.79	8044	12.05	18	−48887.48	.84
	Parameter Estimates for Best Fitting Bivariate Models						
	Best Fitting Model	Bivariate *a* ^2^	Bivariate *c* ^2^	Bivariate *e* ^2^	*r*_a_	*r*_c_	*r*_e_
Paranoia	ACE dropped *r*_c_	.93 (0.85, 1.00)	—	.07 (−0.02, 0.15)	.55 (0.45, 0.70)	—	.04 (−0.01, 0.09)
Paranoia controlling for anxiety	ACE dropped *r*_c_	.92 (0.83, 1.00)	—	.08 (−0.01, 0.17)	.54 (0.43, 0.69)	—	.04 (−0.01, 0.09)
Paranoia controlling for depression	ACE dropped *r*_c_	.93 (0.83, 1.00)	—	.07 (−0.03, 0.17)	.52 (0.40, 0.67)	—	.04 (−0.01, 0.08)
Paranoia controlling for anxiety and depression	ACE dropped *r*_c_	.92 (0.83, 1.00)	—	.08 (−0.03, 0.17)	.52 (0.40, 0.67)	—	.04 (−0.01, 0.08)
Paranoia controlling for bullying victimization age-16	ACE dropped *r*_c_	.89 (0.62, 1.00)	—	.11 (−0.17, 0.38)	.33 (0.19, 0.49)	—	.03 (−0.04, 0.10)

*Note*: Abbreviations are explained in the first footnote to table 4. Bivariate genetic (Bivariate *a* ^2^), common environment (Bivariate *c* ^2^), and unique environment (Bivariate *e* ^2^) estimated indicate the proportion of phenotypic correlations explained by genetics, common, and unique environments, respectively. Bivariate genetic (*r*_a_), common environment (*r*_c_), and unique environment (*r*_e_) correlations indicate the genetic and environmental overlap between psychotic experiences and bullying victimization. 95% confidence intervals in parentheses.

^a^Best fitting model.

In addition, phenotypic analyses demonstrated that the bivariate association between Paranoia and bullying victimization was not significantly confounded by the effects of cannabis use and personality at age of 16 years (see supplementary table 2)

## Discussion

### Childhood Bullying Victimization and Paranoia

This is the first study to investigate whether particular PEs (including positive, cognitive, and negative dimensions) within the general population are associated with childhood bullying victimization in the community, and the relative influences of genes and environment on the association between them. Childhood bullying victimization was most strongly, although modestly associated with adolescent Paranoia, explaining just ~6% of variance in Paranoia 4 years later. Childhood bullying victimization was less strongly, although still significantly associated with Hallucinations, Cognitive Disorganization, and parent-rated Negative Symptoms, explaining ~1%–3% of variance in these later PEs. Childhood bullying victimization was barely associated at all with Anhedonia and Grandiosity. These findings emphasize the value of exploring associations with specific PEs, rather than assuming they can be clumped together or limiting investigations to only some forms of PEs. Our results confirm earlier reports from longitudinal population-based studies in children and adolescents that bullying victimization is a risk factors for PEs,^2,4,8^ and extend these findings by suggesting that it is particularly linked to later Paranoia. If these findings from a young community sample extend to clinical symptomatology in adults, they predict that individuals with a history of bullying victimization will be particularly prone to paranoid symptoms.

Our observations of an association between bullying victimization and PEs are consistent with cognitive psychological theories of the development of PEs.^19^ Empirical evidence showing associations between bullying victimization and negative attributional styles have found that children and young adults who had been bullied in childhood reported more negative attributional styles in comparison with those who had not been bullied and, consequently, were likely to view their environment to be hostile and threatening.^20,21^ This heightened perception of threat and hostility may trigger PEs, such as Paranoia, and help explain the reported association between bullying victimization and Paranoia. Our data are also in line with neurobiological models of psychosis, which propose that subcortical dopamine dysfunction plays a critical role. Recent neuroimaging work suggests that traumatic experiences in childhood are linked to altered striatal dopamine function in adulthood.^34^

### Heritability of Childhood Bullying Victimization

Heritability estimates for bullying victimization indicated approximately one-third (35%) of individual differences were due to genetic factors, with the remainder being explained by environmental factors. It can seem counterintuitive at first that an experience, such as being bullied, is partly heritable. However, risk of bullying victimization is known to be influenced by characteristics in the child who is bullied, such as temperament^35^ and self-esteem,^36^ which are themselves heritable.^37,38^ For example, a child’s temperament where there is a lack of control such as being overly emotional, may evoke negative peer interactions such as bullying victimization. To our knowledge heritability estimates for bullying victimization have been calculated earlier only among one other UK-based sample of children and adolescents.^11^ Our heritability estimate of 35% was lower than that reported by others (73%).^11^ One possible explanation for this discrepancy may lie in the method of measurement. It is possible that our measure, which was a continuous scale and specifically questioned different types of bullying behaviors, such as social manipulation and physical victimization, captured greater individual unique environmental variances in bullying behaviors in contrast to Ball and colleagues,^11^ where severity of bullying victimization was the focus. Secondly, in contrast to Ball and colleagues,^11^ where mothers’ reports of bullying victimization at the age of 9–10 years were collected, we used a self-report measure at the age of 12 years. It is possible that the higher heritability estimates reported by Ball and colleagues^11^ may have been inflated due to shared methods variance as the same parent reported on the bullying victimization experiences of both twins within a twin pair. Genetic factors may also play a less prominent role in the exposure to different types of bullying victimization behaviors an individual may be exposed to in comparison to the severity of bullying victimization. Furthermore, at the age of 12 years when children are making a pivotal transition from primary to secondary school, exposures to new environments (eg, school locker rooms) may become more prominent in influencing their risk of being bullied than at younger ages.

### Genetic Overlap Between Childhood Bullying Victimization and Paranoia

The strongest association found, between childhood bullying victimization and Paranoia, was explained almost in its entirety by shared genetic influences. A considerable proportion of the genes influencing individual differences in childhood bullying victimization overlapped with the genes influencing Paranoia. These findings suggest that in childhood there may be inherent genetic predispositions that orientate children’s behavior and thinking styles in such a way that it makes them jointly vulnerable to being victims of bullies and adopting paranoid thinking styles. This finding is in contrast to the earlier study, whereby the association between bullying victimization was found to be a risk factor for PEs among young adolescents independent of their genetic risk.^2^ However, our study differed by focusing on Paranoia in contrast to Hallucination and Delusions.^2^ It is thus possible that the role of genetic influences may be more prominent in explaining the association with bullying for some forms of PEs (ie, Paranoia) than for others.

Young adolescents may possess a genetic propensity that contributes to being in situations where the risk of bullying victimization is heightened and subsequently increases the risk for PEs. Longitudinal studies have found early emotional problems to increase the risk of bullying victimization.^39^ Anxious and depressed children and adolescents may send signals of difficulties in being able to negotiate conflicts or stand up for themselves and thus be viewed as easy targets for threats and abuse from their peers. As emotional problems are in part heritable^40^ and associated with PEs,^41^ it is theoretically possible that any genetic influences underlying the relationship between bullying victimization and PEs are explained by co-occurring emotional problems.^4^ We explored this in our data and found that the phenotypic and genetic association between bullying victimization and Paranoia remained after controlling for emotional problems at the age of 12 years. Specifically, 92% of the covariation between Paranoia and bullying victimization was explained by genetic influences when anxiety and depression was controlled for.

Furthermore, these data showed that the phenotypic association between bullying victimization and Paranoia remained after controlling for a cannabis use and personality (see supplementary table 2). Overall, these findings provide evidence against these confounders as underlying pathways explaining the covariation between bullying victimization and Paranoia.

The next step is to understand the mechanism underlying the association between bullying victimization and Paranoia. One candidate is negative attribution style. Studies report an association between negative attribution styles and bullying victimization.^20,21^ As attributional styles are in part heritable^42^ and associated with PEs,^43^ genetic influences underlying the relationship between bullying victimization and Paranoia may in part be explained by attribution styles. Such analyses are beyond the scope of our study and warrant further investigation.

### Limitations and Strengths

Although we tested a longitudinal association between bullying victimization at the age of 12 years and PEs at the age of 16 years, we were not able to control for the level of PEs at the age of 12 years. Second, we used self-reports of Paranoia; it is therefore possible that the Paranoia being captured is not unfounded but rather a manifestation of bullying behaviors (ie, “they are out to get me”). This study could be replicated using interviews and reports from other informants. However, because the association between bullying victimization and Paranoia remained after controlling bullying victimization for the age of 16 years, we were able to conclude that the association between bullying victimization at the age of 12 years and Paranoia was not being driven by a continuation of bullying victimization. Lastly, we used self-reports of bullying victimization at the age of 12 years, and it may be questioned whether young adolescents at the age of 12 years are able and comfortable in reporting on such negative experiences; however, self-reports of bullying victimization at the age of 12 years have been found to be reliable and comparable to parent reports.^44^ Further to its limitations, this study’s strengths lie in its genetically informative study twin design, which decomposed the relationship between bullying victimization and Paranoia into genetic and environmental influences. Moreover, the large sample size and multiple dimensions of PEs allowed us to investigate bullying victimization in relation to specific PEs, which included positive, cognitive, and negative PEs.

## Conclusions

This study found bullying victimization in late childhood to be associated most strongly, although modestly with Paranoia, explaining 6% of variance in Paranoia assessed 4 years later in adolescence. Smaller associations were seen between bullying victimization and Hallucinations, Cognitive Disorganization, parent-rated Negative Symptoms (explaining 1%–3% variance), and only negligible associations were present with Grandiosity and Anhedonia. As such, bullying victimization appears to co-occur with later thinking styles of unfounded and excessive fears about others. Although modest, this association was driven almost completely by genetic risk factors that are common to both bullying victimization and Paranoia. Instead of viewing childhood bullying victimization as a purely environmental experience that can trigger later PEs, these findings focus the spotlight on inherited characteristics that may make individuals jointly vulnerable to be bullied and feeling paranoid. Our additional analyses showed that this relationship was not explained by underlying emotional problems at the age of 12 years.

## Supplementary Material

Supplementary material is available at http://schizophreniabulletin.oxfordjournals.org.

## Funding

Medical Research Council (G1100559 to A.R.; G0901245, G0500079 to R.P.; G0902308 to D.F.).

## Supplementary Material

Supplementary Data
