# Supplementary material for: A Shared Genetic Propensity Underlies Experiences of Bullying Victimization in Late Childhood and Self-Rated Paranoid Thinking in Adolescence
Source: Schizophr Bull. 2014 Oct 16;41(3):754–63. doi: 10.1093/schbul/sbu142 (PMC4393686; doi:10.1093/schbul/sbu142)
Supplement: Supplementary Data [file supp_41_3_754__index.html]

A Shared Genetic Propensity Underlies Experiences of Bullying Victimization in Late Childhood and Self-Rated Paranoid Thinking in Adolescence — Supplementary Data 

# A Shared Genetic Propensity Underlies Experiences of Bullying Victimization in Late Childhood and Self-Rated Paranoid Thinking in Adolescence

## Supplementary Data

Data files

**Files in this Data Supplement:**

- Supplementary Data - Supplementary Data
