# Supplementary material for: A Shared Genetic Propensity Underlies Experiences of Bullying Victimization in Late Childhood and Self-Rated Paranoid Thinking in Adolescence
Source: Schizophr Bull. 2014 Oct 16;41(3):754–63. doi: 10.1093/schbul/sbu142 (PMC4393686; doi:10.1093/schbul/sbu142)
Supplement: Supplementary Data [file supp_sbu142_SHAKOOR_Resubmission_Shakoor_Bullying_PEs_Supplementary_28August14.pdf]

Supplementary material

Table 1: Intraclass Twin Correlations split by gender

	MZ	DZ
	ICC (CI)	ICC (CI)
Total sample		
Univariate twin correlations		
Paranoia	0.52 (0.49, 0.56)	0.29 (0.24, 0.33)
Bullying victimization	0.62 (0.58, 0.65)	0.42 (0.37, 0.46)
Cross-trait cross-twin correlation		
Paranoia and bullying victimization	0.26 (0.21, 0.31)	0.12 (0.07, 0.18)
Males		
Univariate twin correlations		
Paranoia	0.48 (0.42, 0.53)	0.27 (0.20, 0.34)
Bullying victimization	0.59 (0.54, 0.65)	0.43 (0.36, 0.49)
Cross-trait cross-twin correlation		
Paranoia and bullying victimization	0.25 (0.17, 0.33)	0.12 (0.03, 0.20)
Females		
Univariate twin correlations		
Paranoia	0.55 (0.51, 0.59)	0.30 (0.24, 0.36)
Bullying victimization	0.63 (0.59, 0.67)	0.41 (0.35, 0.47)
Cross-trait cross-twin correlation		
Paranoia and bullying victimization	0.27 (0.21, 0.33)	0.13 (0.06, 0.20)

Note: Intraclass correlations using transformed standardized age and sex regressed scales. ICC= Intraclass correlations CI= confidence intervals.

Table 2: Paranoia age-16 and bullying victimization age-12: Phenotypic correlations adjusting for confounders

	Paranoia<br>**r (95%CI)**
Unadjusted bullying victimization age -12	0.26 (0.23, 0.28)
Anxiety age-12	0.24 (0.22, 0.27)
Depression age-12	0.23 (0.20 0.25)
Bullying victimization age-16	0.14 (0.09, 0.18)
Cannabis use age-16	0.25 (0.22, 0.28)
Neuroticism age-16	0.21 (0.17, 0.25)
Extraversion age-16	0.27 (0.23, 0.31)
Openness age-16	0.28 (0.24, 0.31)
Agreeableness age-16	0.27 (0.23, 0.31)
Conscientiousness age-16	0.27 (0.23, 0.31)
Unadjusted bullying victimization age-12	0.26 (0.23, 0.28)

Note: Correlations were performed using one random member of each twin pair using standardized age and sex regressed residuals. *r* = Pearson's correlation, CI= confidence intervals.
